# Perceived fatigue is highly prevalent and debilitating in patients with mitochondrial disease

**DOI:** 10.1016/j.nmd.2015.03.001

**Published:** 2015-07

**Authors:** Gráinne S. Gorman, Joanna L. Elson, Jane Newman, Brendan Payne, Robert McFarland, Julia L. Newton, Douglass M. Turnbull

**Affiliations:** a Wellcome Trust Centre for Mitochondrial Research, Institute for Ageing and Health, Newcastle University, Newcastle upon Tyne NE2 4HH, UK; b Institute of Ageing and Health and NIHR Biomedical Research Centre for Ageing, Newcastle University, Newcastle upon Tyne NE4 5PL, UK; c Institute of Genetic Medicine, Newcastle University, Newcastle upon Tyne NE1 3BZ, UK

**Keywords:** Mitochondria, Fatigue, Depression, Anxiety, Sleep

## Abstract

Perceived fatigue is a prominent symptom in patients with mitochondrial disease but to date its prevalence, impact and aetiology are poorly understood. Our aim was to determine the prevalence and assess for comorbidities associated with clinically relevant fatigue in patients with mitochondrial disease. A cross-sectional postal survey of patients with mitochondrial disease was undertaken using a validated self-completion, patient-reported outcome measures (response rate: 60%; n = 132). The prevalence and perceived functional impact of experienced fatigue were assessed using the Fatigue Impact Scale. Other putative biological mechanisms were evaluated using the Hospital Anxiety Depression scale and Epworth sleepiness scale. Data were compared with those for healthy control subjects and patients with Myalgic Encephalopathy/Chronic Fatigue Syndrome matched for age and gender. Sixty-two per cent of patients with mitochondrial disease reported excessive symptomatic fatigue (Fatigue Impact Scale ≥ 40); whilst 32% reported severe, functionally limiting fatigue symptoms (Fatigue Impact Scale ≥ 80) comparable to perceived fatigue in patients with Myalgic Encephalopathy/Chronic Fatigue Syndrome. Fatigue is common and often severe in patients with mitochondrial disease irrespective of age, gender or genotype. Future evaluation of causal factors in mitochondrial disease-associated fatigue is warranted with the potential to guide future treatment modalities.

## Highlights

• Perceived fatigue is a prominent and often debilitating symptom in patients with mitochondrial disease.
• Perceived fatigue correlates with disease severity but not genotype.
• Excessive sleepiness is prevalent but dissociated with perceived fatigue in patients with mitochondrial disease.
• Perceived fatigue does not correlate with NMDAS muscle weakness scores.
• Our findings have important implications for targeting of pharmacological therapies.

## 1 Introduction

Perceived fatigue is a prominent and often debilitating symptom in patients with mitochondrial disease yet the prevalence, impact and aetiology of fatigue are poorly understood. From an operational perspective, we define *perceived fatigue* as an overwhelming sense of tiredness, lack of energy or feeling of exhaustion [1] employing a multifactorial approach; distinguishing this from *physiological fatigue* in which the focus is muscle and its ability to generate and maintain power. The premise of this study was to determine the magnitude and impact of *self-perceived* fatigue in a large, genetically heterogeneous group of patients with mitochondrial disease, whilst evaluating putative biological mechanisms that have been recognised in other neurological disorders and chronic disease states.

### 1.1 Participants and methods

A postal survey of 215 adult patients with mitochondrial disease attending our specialist outpatient clinic was undertaken. Demographic data and disease burden established using the Newcastle Mitochondrial Disease Adult Scale (NMDAS) [2] were retrieved from clinical records. Myalgic Encephalopathy/Chronic Fatigue Syndrome (CFS/ME) patients (n = 74), as defined by the Centers for Disease Control and Prevention 1994 Fukuda criteria [3], attending a specialist out-patient care service were surveyed. Healthy control subjects (n = 132) were Control subjects recruited through notices in local press and hospitals asking for volunteers to participate in research projects, with no selection made for the presence or absence of fatigue and were matched for age and gender. Institutional ethical approval was obtained.

### 1.2 Symptom assessment tools

#### 1.2.1 Assessing fatigue

The presence and perceived impact of fatigue was assessed using the Fatigue Impact Scale (FIS) [4], a validated global measure of fatigue comprising 40 questions covering three domains: cognitive, physical and psychosocial. FIS scores reflect the perceived functional impact of experienced fatigue on these domains within the previous one month [5]. A score of ≥40 indicates excessive symptomatic fatigue and ≥80 severe, symptomatic fatigue. To date, FIS has been validated for several chronic disease states in which fatigue is recognised as a severe, debilitating symptom including CFS/ME, multiple sclerosis, chronic obstructive pulmonary disease, primary biliary sclerosis and chronic hepatitis C [5].

#### 1.2.2 Assessing for covariates of perceived fatigue

All participants completed the Hospital Anxiety Depression scale (HAD) [6] to assess for depression (HAD-D) and anxiety symptoms (HAD-A), and the Epworth sleepiness scale (ESS) [7] to qualitatively measure daytime sleepiness. Disease burden was established using the Newcastle Mitochondrial Disease Adult Scale (NMDAS) [2].

### 1.3 Statistical analyses

All statistical analyses were performed using SPSS version 19. Chi square analysis was used to determine gender differences in the study groups. The comparisons of FIS, HAD, and ESS between the three groups were conducted using a One Way analysis of variance (ANOVA), with a Tukey post-hoc test. One Way ANOVA was also used to examine if genotype had an impact on fatigue in patients with mitochondrial disease. Multiple stepwise regression was used to determine whether covariates analysed were significant predictors of fatigue.

## 2 Results

### 2.1 Patient and control characteristics

Of the initial 215 patients with mitochondrial disease surveyed, one hundred and thirty-two questionnaires were completed and returned (response rate: 60%) (Table 1): 91 females; mean age 52 years old (range 18–82); mean NMDAS score 27 (±18). Control subjects (mean age 52 years; range 21–77) were age and gender matched for patients with mitochondrial disease. Although CFS/ME patients (mean age 54 years; range 24–80) were age-matched; there were fewer men (24%); however, the gender ratios between all three groups were not significantly different (Chi square p = 0.401).

### 2.2 Prevalence and impact of fatigue

**Fig. 1:**
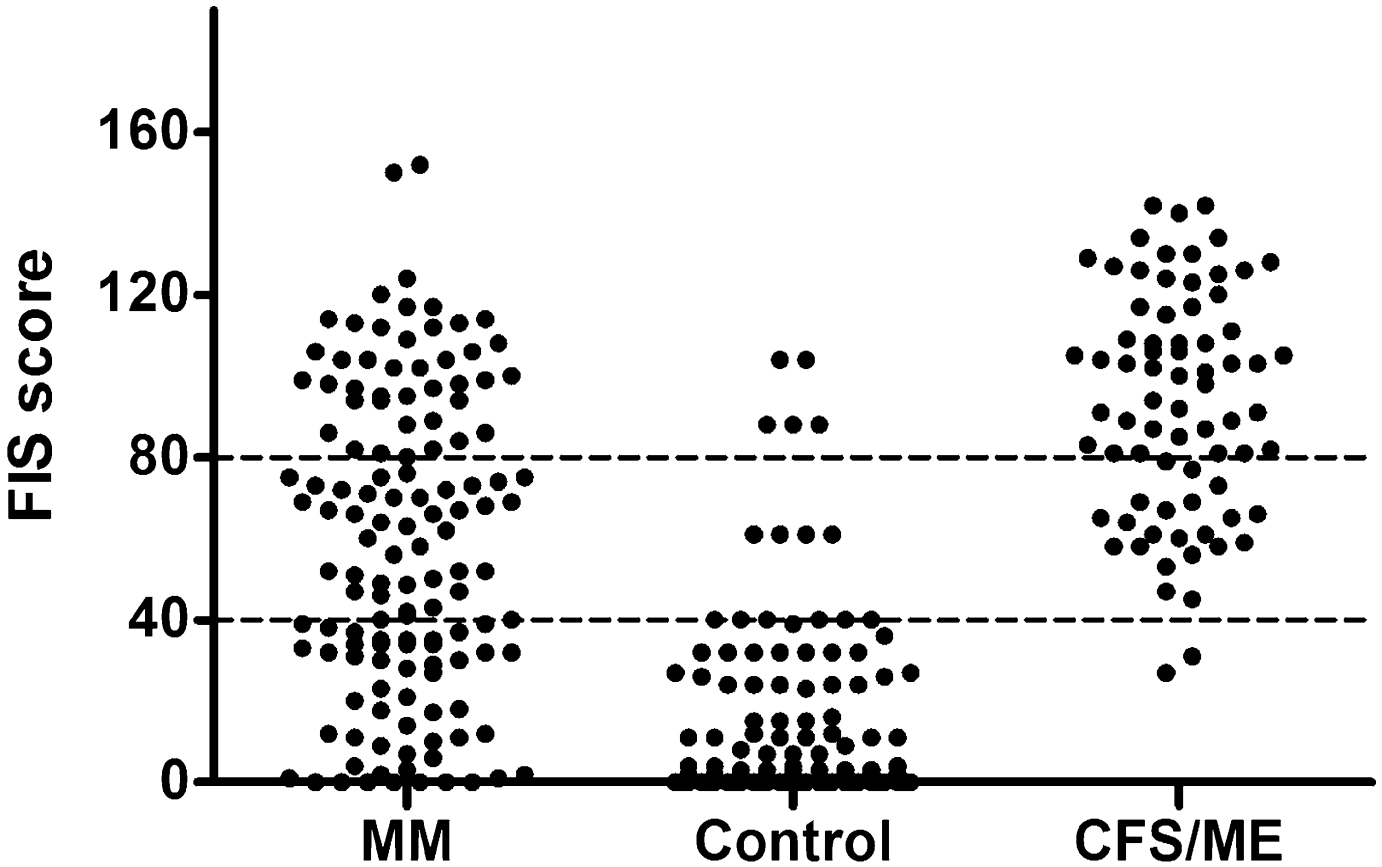
Distribution of Fatigue Impact severity (FIS) scores in patients with mitochondrial disease (MM), healthy control volunteers (control); p < 0.001 and chronic fatigue syndrome/myalgic encephalomyelitis (CFS/ME) patients. FIS ≥ 40 indicates excessive symptomatic fatigue; FIS ≥ 80 indicates severe symptomatic fatigue experienced in the past one month.

Fatigue was common in patients with mitochondrial disease (mean FIS 58; SD 38) with 62% of patients reporting excessive symptomatic fatigue (FIS ≥ 40) (Table 2). The mean FIS scores among control subjects were 13 (SD 22) and 92 (SD 28) in CFS/ME patients. The highest FIS scores in patients with mitochondrial disease were comparable to those in patients with CFS/ME (Fig. 1). The magnitude of symptomatic fatigue and its perceived impact in patients with mitochondrial disease correlated with disease burden (Pearson's correlation co-efficient r = 0.326, p < 0.0001), but was independent of genotype (one-way between groups ANOVA; F (6,125) = 0.49, p = 0.9). Using Spearman Rho (after Bonferroni correction) the NMDAS domains that significantly correlated with FIS were swallowing, cutting food, dressing, hygiene, exercise tolerance, gait and psychiatric. Notably all other domains were not significant including myopathy (Supplementary Table S1). Using each of the reported NMDAS myopathy scores (as groups) and FIS scores in a one-way ANOVA, there was no difference found between the myopathy categories (ANOVA, p = 0.16) suggesting that fatigue is not an outcome of muscle weakness score. There was no significant difference in the impact of fatigue between men (independent-samples t-test, M = 45.10, SD = 43.71) and women (M = 49.60, SD = 43.02; *t* (336) = 0.881, p = 0.34) between the three groups.

### 2.3 Anxiety, depression and daytime sleepiness

Mean anxiety (HAD-A) scores were similar in both chronic disease states (patients with mitochondrial disease; 8 (SD 5); CFS/ME patients 8 (SD 5); control subjects; 5 (SD4)). There was no significant difference in anxiety symptoms (HAD-A scores) between patients with mitochondrial disease and CFS/ME (One Way ANOVA, Tukey's post-hoc test F (2,334) = 17.392, p < 0.983); control subjects were significantly different from both disease groups, overall ANOVA value (F (2,334) = 17.392, p < 0.0001). Depressive symptoms (HAD-D) were less prevalent in patients with mitochondrial disease (mean 5; SD 4) compared to CFS/ME patients (mean 8; SD 4), but higher than control subjects (mean 2; SD 3); one-way between groups ANOVA (F (2,334) = 60.885, p < 0.0001). Twenty-seven per cent of patients with mitochondrial disease reported excessive daytime somnolence as assessed by ESS scores greater than 10 (mean ESS 6; SD 5). However, there was no significant difference between ESS scores for all three groups (one-way between groups ANOVA; F (2,335) = 1.65; p = 0.2).

### 2.4 Predictive model of fatigue

HAD-A, ESS, group, and HAD-D were retained as significant predictors of FIS, whilst age and gender were not significant predictors across the three groups. HAD-A, ESS, group, and HAD-D accounted for half of the variance in fatigue across all the participants who took part in the study (Stepwise-regression analysis, adjusted R^2^ = 0.504), with a model significance given as p < 0.001 (F (4,335) = 86.501).

## 3 Discussion

Kluger et al. [8] have recently proposed a unified taxonomy for the evaluation of fatigue that provides a practical strategy for conducting research of this common and often neglected aspect of mitochondrial disease. Implementing this framework, the aims of this study were to determine the prevalence and nature of perceived fatigue in a large, genetically heterogeneous group of patients with mitochondrial disease and systematically assess potential covariates. We demonstrate that clinically relevant fatigue is common and is often perceived to severely impact on functionality in patients with mitochondrial disease irrespective of age, gender and perhaps most surprisingly genotype. Both the extent of fatigue experienced and its impact in mitochondrial subjects were significantly greater than in the age and gender matched control group. Sixty-two percent of mitochondrial patients surveyed reported significant symptomatic fatigue (FIS ≥ 40) with 32% reporting severe symptomatic fatigue (FIS ≥ 80) with psychosocial and physical domains more affected (p < 0.0001). Interestingly, although there were no significant differences between male and female patients with mitochondrial disease, in reported symptomatic fatigue, male participants tended to report higher functional limitation due to experienced fatigue which may be counter intuitive and contrary to other chronic diseases in which fatigue is a prominent symptom.

Prior to this study, although patients had been identified clinically as having symptoms of fatigue, few had been recognised to have such prominent fatigue comparable to that in patients with CFS/ME. Potential bias may have arisen in that respondents may have been only those with significant fatigue; however, respondent disease burden and demographics are strikingly representative of our patient cohort as a whole. Hence it is likely that the severity and prevalence of fatigue symptoms is decidedly under-recognised in patients with mitochondrial disease.

Multiple factors have been implicated in influencing the perception of fatigue including psychological parameters, sleep disturbance and central and peripheral neurological dysfunction [9]. Regression analysis suggested that anxiety, depression and daytime sleepiness may be confounding factors of perceived fatigue in patients with mitochondrial disease. However given the high prevalence of fatigue in the absence of clinically relevant anxiety or depression, in our patient cohort, it is unlikely that the main driver of fatigue relates primarily to a mood disorder. Similarly, although a third of those surveyed scored greater than 10 on ESS, overall scores were not different in patients with mitochondrial disease compared to control subjects suggesting a clear dissociation between poor sleep hygiene and perceived fatigue. Moreover, the ability of disease burden to predict the presence and impact of fatigue in patients with mitochondrial disease suggests that if we were able to improve the biochemical defect or stop disease progression, perceived fatigue would be less, irrespective of the underlying fatigue mechanisms.

NMDAS domains that correlated with FIS were predominantly activities of daily living (Supplementary Fig. S1) reflecting the nature of the assessment tool employed (FIS), namely, functional limitation due to experienced fatigue, and the prevalence of symptoms in the disease group (patients with mitochondrial disease) evaluated. The severity of encephalopathy as defined by encephalopathy NMDAS sub score was not associated with fatigue (Supplementary Table S1). This is perhaps not surprising as stroke-like episodes and the ensuing encephalopathy which are recognised, devastating consequences of mitochondrial disease, affect only a relatively small (10–15%) proportion of individuals who have inherited the m.3243A>G mutation in mitochondrial DNA which is the most common pathogenic point mutation in mitochondrial DNA [10]. In addition myopathy did not correlate with FIS and may reflect the low prevalence of myopathic symptoms as assessed by NMDAS in this cohort of patients. However, we concede that this cohort of patients evaluated exhibit marked genotypic and phenotypic variability that may impact on current findings particularly in relation to the absence of correlation of FIS with genotype and propose that a future study with greater numbers with more rare mitochondrial mutations may help correct for this potential bias.

To our knowledge, this series is the largest prospective, patient-reported outcome measure study to quantify the prevalence and symptom severity of perceived fatigue and evaluate possible covariates in patients with variable genotypic mitochondrial disease. Although we have not assessed the secondary impact of medications due to the low numbers involved, important conclusions may be drawn. Fatigue is common, frequently severe, and similar in magnitude to that reported in other chronic neurological disorders [8]. Sleep impairment can readily be distinguished from perceived fatigue arising as a primary manifestation of mitochondrial disease whilst there is clearly a more complex association between perceived fatigue and mood disorders, warranting further assessment.

The challenge now is to identify causal factors that may help direct tailored pharmacological and non-pharmacological symptomatic therapeutic strategies, including those that may ameliorate the biochemical defect or halt progression; with potential for a shared therapeutic paradigm with patients with other chronic neurological disorders, exhibiting clinically relevant fatigue.

## Figures and Tables

**Table 1:** Summary table of genotypes of 132 respondents with mitochondrial disease.

Genotype	Number
mt-tRNA mutations	
m.3243A>G MELAS mutation	39
m.8344A>G MERRF mutation	9
LHON	2
NARP	1
Others	12
Single-large scale mt-DNA deletion	26
Multiple mtDNA deletions	
OPA1	3
POLG1	11
C10orf2	12
RRM2B	3
SPG7	1
Unspecified nuclear genetic defect	13

MELAS: mitochondrial encephalopathy, lactic acidosis and stroke-like episodes; MERRF: myoclonic epilepsy with ragged red fibres; LHON: Leber's hereditary optic neuropathy; NARP: neuropathy, ataxia and retinitis pigmentosa; mt: mitochondrial; tRNA: transfer RNA; OPA1: optic atrophy type 1; POLG1: polymerase gamma; C10orf2: chromosome 10 open reading frame 2; RRM2B: ribonucleotide reductase M2B (TP53 inducible); SPG7: spastic paraplegia 7 gene mutations.

**Table 2:** The rank frequency of fatigue severity as assessed by FIS (Fatigue Impact Scale).

FIS Rank	FIS descriptive	FIS scoring	Results (n=)
0	No fatigue	0–10	16
1	Mild	10–37	31
2	Moderate	38–79	43
3	Severe	80–119	38
4	Very severe	129–160	4

## Appendix Supplementary material

Fig. S1 Fatigue Impact Scale (FIS) correlates with disease burden (NMDAS) in patients with mitochondrial disease.

Table S1 Assessing the impact of disease burden (NMDAS) on perceived fatigue (FIS): NMDAS domains of swallowing, cutting food, dressing, hygiene, exercise tolerance, gait and psychiatric correlate with FIS scores, using Spearman Rho (after Bonferroni correction). Notably all other NMDAS domains including myopathy do not significantly correlate with perceived fatigue.
