# Supplementary figures and images for: Perceived fatigue is highly prevalent and debilitating in patients with mitochondrial disease
Source: Neuromuscul Disord. 2015 Jul;25(7):563–6. doi: 10.1016/j.nmd.2015.03.001 (PMC4502433; doi:10.1016/j.nmd.2015.03.001)

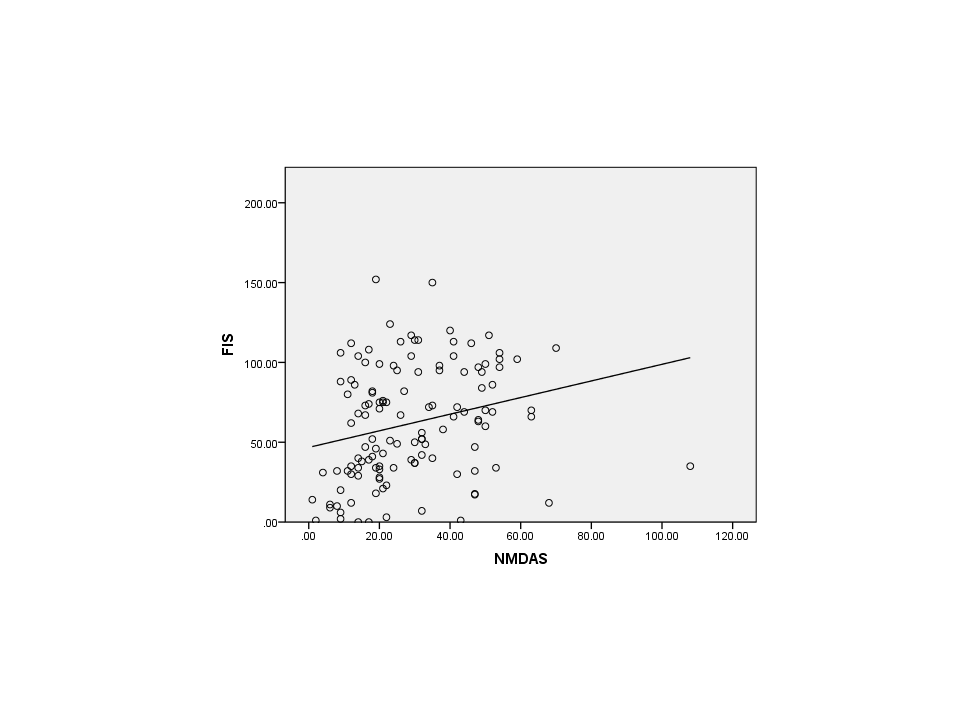

Supplement: Fig. S1 — Fatigue Impact Scale (FIS) correlates with disease burden (NMDAS) in patients with mitochondrial disease. [file mmc1.zip › mmc1.tif]
