# Supplementary material for: Perceived fatigue is highly prevalent and debilitating in patients with mitochondrial disease
Source: Neuromuscul Disord. 2015 Jul;25(7):563–6. doi: 10.1016/j.nmd.2015.03.001 (PMC4502433; doi:10.1016/j.nmd.2015.03.001)
Supplement: Table S1 — Assessing the impact of disease burden (NMDAS) on perceived fatigue (FIS): NMDAS domains of swallowing, cutting food, dressing, hygiene, exercise tolerance, gait and psychiatric correlate with FIS scores, using Spearman Rho (after Bonferroni correction). Notably all other NMDAS domains including myopathy do not significantly correlate with perceived fatigue. [file mmc2.docx]

|  | Vision | Hearing | Speech | Swallow | Handwriting | Cutting | Dressing | Hygiene | Exercise | Gait |
| --- | --- | --- | --- | --- | --- | --- | --- | --- | --- | --- |
| Correlation Coefficient | 0.063 | 0.111 | 0.224 | 0.326 | 0.112 | 0.273 | 0.344 | 0.307 | 0.389 | 0.27 |
| sig (2-tailed) uncorrected | 0.482 | 0.211 | 0.011 | 1.74E-04 | 0.17 | 0.002 | 7.10E-05 | 4.32E-04 | 5.83E-06 | 0.02 |
| sig (2-tailed) corrected |  |  | 0.297 | 4.69E-03 |  | 0.054 | 1.92E-03 | 1.17E-02 | 1.57E-04 | 0.54 |
|  |  |  |  |  |  |  |  |  |  |  |
|  | Psychiatric | Migraine | Seizures | Stroke-like | Encephalopathic | GIT | Diabetes | Respiratory | Cardiovascular | Visual |
| Correlation Coefficient | 0.353 | 0.249 | 0.067 | 0.001 | 0.077 | 0.192 | 0.01 | 0.103 | 0.082 | -0.12 |
| sig (2-tailed) uncorrected | 4.40E-05 | 0.005 | 0.452 | 0.987 | 0.387 | 0.03 | 0.912 | 0.251 | 0.363 | 0.179 |
| sig (2-tailed) corrected | 1.19E-03 | 0.135 |  |  |  | 0.81 |  |  |  |  |
|  |  |  |  |  |  |  |  |  |  |  |
|  | Ptosis | PEO | Dysphonia/dysarthria | Myopathy | Cerebellar | Neuropathy | Pyramidal | Extrapyramidal | Cognition |  |
| Correlation Coefficient | 0.056 | 0.003 | 0.077 | 0.218 | 0.214 | 0.07 | 0.147 | -0.024 | n/a |  |
| sig (2-tailed) uncorrected | 0.53 | 0.976 | 0.391 | 0.014 | 0.016 | 0.439 | 0.104 | 0.792 | n/a |  |
| sig (2-tailed) corrected |  |  |  | 0.378 |  |  |  |  |  |  |
|  |  |  |  |  |  |  |  |  |  |  |

Supplemental table 1

Sig=significant; GIT= gastrointestinal tract; PEO= progressive external ophthalmoplegia
